# Evaluation of the Possibility of Using 1.4462 and 1.4501 Steel as a Construction Material for Apparatus Operating at an Increased Temperature and with Corrosive Factors

**DOI:** 10.3390/ma14144014

**Published:** 2021-07-18

**Authors:** Karol Prałat, Andżelika Krupińska, Marek Ochowiak, Sylwia Włodarczak, Magdalena Matuszak, Justyna Ciemnicka, Artur Koper, Karolina Wójcicka

**Affiliations:** 1 Faculty of Civil Engineering, Mechanics and Petrochemistry, Warsaw University of Technology, I. Łukasiewicza 17, 09-400 Płock, Poland; Justyna.Ciemnicka@pw.edu.pl (J.C.); Artur.Koper@pw.edu.pl (A.K.); Karolina.Wojcicka@pw.edu.pl (K.W.); 2 Department of Chemical Engineering and Equipment, Poznan University of Technology, Pl. M. Sklodowska-Curie 5, 60-965 Poznan, Poland; andzelika.krupinska@put.poznan.pl (A.K.); marek.ochowiak@put.poznan.pl (M.O.); sylwia.wlodarczak@put.poznan.pl (S.W.); magdalena.matuszak@put.poznan.pl (M.M.)

**Keywords:** duplex stainless steels, corrosion, chemical apparatus, construction material, sigma phase

## Abstract

The objective of this study was to determine the requirements for steels used as construction materials for chemical apparatus operating at an elevated temperature and to correlate them with the properties of the tested steels. The experimental part examined the influence of the annealing process on the structure and properties of X2CrNiMoN22-5-3 (1.4462) and X2CrNiMoCuWN25-7-4 (1.4501) steel. Heat treatment was carried out on the tested samples at a temperature of 600 °C and 800 °C. Changes were observed after the indicated time intervals of 250 and 500 h. In order to determine the differences between the initial state and after individual annealing stages, metallographic specimens were performed, the structure was analyzed using an optical microscope and the micro-hardness was measured using the Vickers method. Potentiostatic tests of the samples were carried out to assess the influence of thermal process parameters on the electrochemical properties of the passive layer. An increase in the hardness of the samples was observed with increasing temperature and annealing time, the disappearance of magnetic properties for both samples after annealing at the temperature of 800 °C, as well as a significant deterioration in corrosion resistance in the case of treatment at a higher temperature.

## 1. Introduction

Fulfillment-determined emission standards are one of the basic aims, but also the problems, e.g., for large industrial plants [1,2]. Solutions are being sought to optimize the operation of the generated exhaust gas cleaning systems. Besides the appropriate selection of the type of installation for a specific application, its operating parameters, and automation, the key stage is the selection of the construction material from which the apparatus is to be made [3]. Boiler flue gas cleaning systems allow not only a reduction in sulfur compounds, but also NO_x_, HF, HCl, heavy metals, and dioxins. Therefore, devices operating in such a system are exposed to an extremely aggressive, corrosive environment, as well as increased temperature. Another important problem related to the emission of pollutants into the air is the use of inadequate boilers [4].

For the production of the installation elements operating at elevated temperatures and with corrosive substances, it is necessary to select materials with a defined specification [5]. Many requirements are imposed on the construction materials, from which the apparatus operating under hot gas flow conditions is made. The most common group of construction materials used in the apparatus are steels. The choice of steel depends on the parameters of the apparatus and the properties of the material that will be inside the apparatus and should be carried out individually [6]. A very important aspect influencing the selection of construction materials is mechanical criteria, related to, e.g., the loads occurring during operation, system pressure, contact geometry, and sliding speed. Moreover, the decisive influence is also exerted by environmental conditions, which include, e.g., temperature or the type of factors with which the device is in contact [7].

Steels used in such systems must have thermal and corrosion resistance, an increased creep limit, adequate toughness, and wide processing options. The last of the conditions results, inter alia, from the fact that boilers and pipes are made of various types of elements—flat sheets as well as spatial sections, pipes, screws or other cables.

The idea of duplex stainless steels combines the best features of ferritic steels with the best properties of austenitic steels [8]. As a result, it is considered one of the most valuable types of steel. Austenitic–ferritic steels are characterized by a structure usually containing 40–50% of austenite in the ferritic matrix, which results from the increased content of Cr and a limited amount of Ni [9]. These steels show satisfactory mechanical properties, which are related to the mechanical properties of ferrite and austenite [10]. The presence of ferrite increases the yield point, as well as hardness and brittleness. Austenite increases the ductility and toughness of the material [11]. Moreover, these steels are characterized by very good corrosion resistance (especially intercrystalline and pitting) and weldability [12]. These features have determined the possibility of their various applications in many industries. They are successfully used in the chemical, shipbuilding, aviation, and cryogenic industries, as well as in the paper, mining, and petrochemical industries. They are used for building structures, storage tanks, chemical tanks, pressure vessels, and process equipment. They can be found as a construction material, e.g., in desalination processes, peroxide reactors, fermentation chambers, expansion tanks, pipes, flexible hoses, domestic heating devices, and hot water tanks [13]. They are good materials for dryers, heat exchangers, structures operating in aggressive environments (e.g., in seawater, H_2_S, CO_2_), and dynamically loaded devices [14].

When analyzing the microstructure of duplex stainless steel, the possibility of the precipitation of intermetallic phases should be remembered [15,16]. The appearance of additional precipitates may cause significant changes in the properties of the material [17]. One of the most dangerous phases is the sigma phase, which deteriorates the resistance of steel to pitting corrosion by limiting the content of Cr and Mo [18]. In addition, properties such as the strength and ductility of the steel deteriorate when precipitation occurs. The formation of the sigma phase is a function of the steel chemical composition, time, and temperature of heat treatment [19]. There are many literature articles on the phenomenon of precipitation of the intermetallic sigma phase for alloy steels [20,21]. However, there is still no clear relationship between the amount of precipitation formed and the conditions contributing to its formation, as well as the effect it exerts on specific properties. Information about what amount of sigma would be acceptable is being sought.

Due to a lot of advantages, duplex stainless steels are eagerly chosen as a construction material for equipment operating in difficult, corrosive conditions resulting from the action of increased temperature and the influence of an aggressive environment. Their features ensure potential savings resulting from the increased durability of the installation.

The purpose of this work was to study the influence of heat treatments on the changes in the microstructure and properties of stainless steels 1.4462 and 1.4501. The correlation was sought between annealing parameters—temperature and time—and between the amount of intermetallic phases and the obtained effect—properties of tested steels.

## 2. Materials and Methods

The tested samples were made of X2CrNiMoN22-5-3 (1.4462) and X2CrNiMoCuWN25-7-4 (1.4501) steel. The sample 1.4462 was in the form of a profile, while that of steel 1.4501—a flat sheet.

In order to determine the influence of heat treatment on the structure and properties of the tested steels, a number of tests were carried out. The first step was to analyze the chemical composition of the tested samples. For this purpose, a scanning electron microscope (Tescan Vega 5135, Tescan, Brno, Czech Republic) equipped with an EDS X-ray (PGT Prism 200 Avalon, Princeton Gamma-Tech, Princeton, NJ, USA) spectrometer was used. Then, annealing was performed independently at two different temperatures of 600 and 800 °C, respectively, for 250 and 500 h. The metallographic samples were made to examine the microstructure of the steel after treatment and to compare it with the microstructure of the specimens before annealing—in the initial state. Using the secant method, a quantitative analysis was performed for the metallographic samples. In this way, the percentage share of individual phases was estimated. Measurement of micro-hardness and potentiostat tests were carried out for samples subjected to all the above-mentioned conditions and in the initial state. The experimental research hardness was carried out on a micro-hardness tester Walter VMHT MOT, model VMH-002VD (Walter Uhl technische Mikroskopie Gmbh & Co. KG, Asslar, Germany). The test was performed at a load of 50 G. For each of the analyzed steels, 10 micro-hardness measurements were made for each phase. 

Potentiostatic polarization studies were carried out using Autolab PGSTAT 302 N potentiostat/galvanostat (nLab sp. z o.o., Warsaw, Poland). The samples were immersed in a 3M aqueous NaCl solution and potentiostatically polarized in +700 mV SCE for 120 s at 25 °C. The reference electrode was a calomel electrode (SCE).

## 3. Results

**Figure 1:**
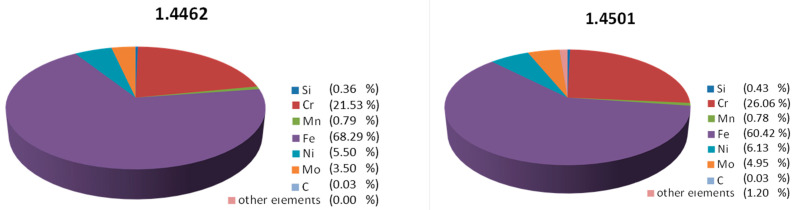
The chemical composition of the tested steels—EDS microanalysis.

The chemical compositions of the analyzed steels are shown in Figure 1 (wt%).

Both samples are high-alloy steels. The qualitative and quantitative differences can be noticed in their composition. The largest quantitative differences concern the elements Cr, Ni, Mo and Fe.

**Figure 2:**
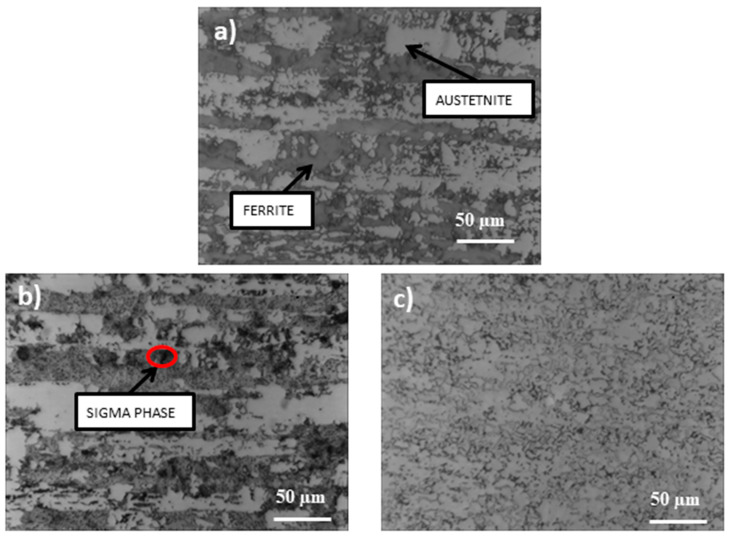
Microstructure of 1.4462 steel: (**a**) before annealing, (**b**) after annealing at 600 °C, (**c**) after annealing at 800 °C.

**Figure 3:**
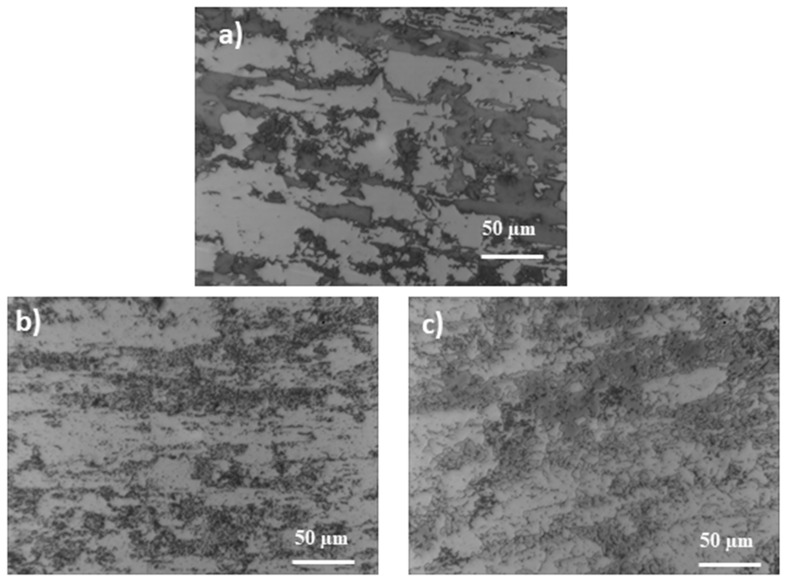
Microstructure of 1.4501 steel: (**a**) before annealing, (**b**) after annealing at 600 °C, (**c**) after annealing at 800 °C.

Figure 2 and Figure 3 show the structures of the X2CrNiMoN22-5-3 (1.4462) and X2CrNiMoCuWN25-7-4 (1.4501) steels in their initial state and after a thermal treatment lasting 250 h.

Both samples show a typical austenitic–ferritic two-phase structure. The bright areas of the images are austenite and the dark areas are ferrite. After annealing at the temperature of 600 °C, for 250 h, apart from the initially present austenite and ferrite, additional σ phase precipitations (formed inside the ferrite) can be seen on them. Heat treatment at higher temperatures causes the dark areas (ferrite phase) to disappear rapidly and the banding of the structure to disappear. In the case of steel 1.4501, the changes in structure occur to a lesser extent.

**Figure 4:**
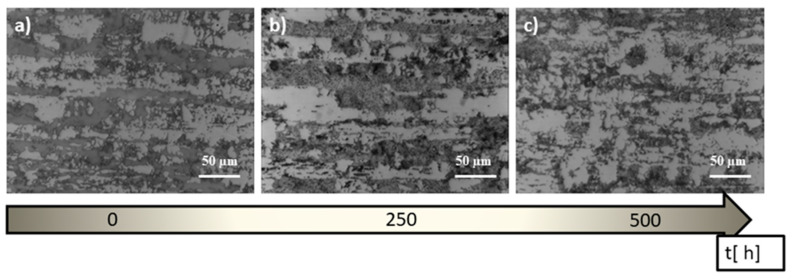
Microstructure of 1.4462 steel: (**a**) before annealing, (**b**) after annealing at 600 °C, 250 h, (**c**) after annealing at 600 °C, 500 h.

A very important parameter of heat treatment is its time duration. Figure 4 shows the changes in the structure of 1.4462 steel during annealing at 600 °C after 250 and 500 h, respectively.

It can be seen that as the duration of the heat treatment progresses, the ferrite further breaks down (reducing the amount of dark areas). Additionally, after long-term annealing, apart from austenite and ferrite, precipitation of the σ phase is visible.

**Figure 5:**
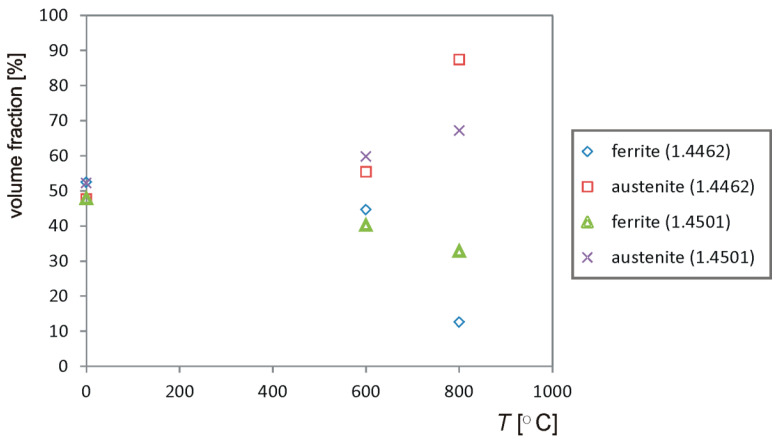
Quantitative analysis of the structure of the tested steels (annealing time—250 h).

It was observed that for both steels the ferrite content decreases with increasing the annealing temperature. During annealing at 800 °C, the ferrite content increases with the time of the heat treatment. The likely cause of this is the precipitation of δ ferrite under these process conditions. Examples of relationships for the tested steels are shown in Figure 5. It should be noted that the share of ferrite and the σ phase was summed up and described as “ferrite”.

**Figure 6:**
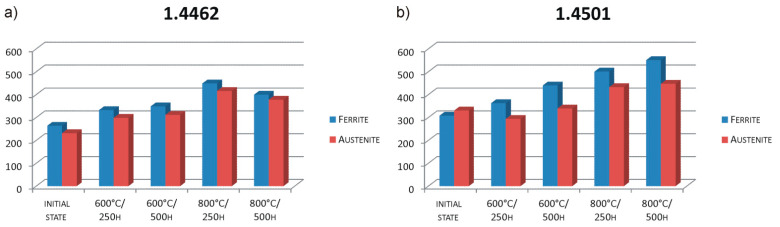
Micro-hardness test results (HV_0.05_): (**a**) X2CrNiMoN22-5-3 (1.4462), (**b**) X2CrNiMoCuWN25-7-4 (1.4501).

The micro-hardness measurement was performed for the tested samples. The averaged results are presented in a comparative way in Figure 6.

When analyzing the obtained data, it can be noticed that with the increase in the annealing time and temperature, the hardness in most cases increases. At each defined test point, 1.4501 steel showed higher hardness.

Macroscopic observations showed that both the samples annealed at 600 and 800 °C lost their magnetic properties after heat treatment.

**Figure 7:**
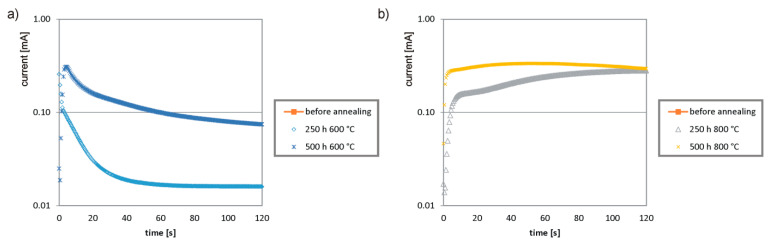
Potentiostatic tests for 1.4462 steel: (**a**) annealing at 600 °C, (**b**) annealing at 800 °C.

The passive layer is responsible for the protection of stainless steels against corrosive factors. In order to assess the effect of heat treatment on the electrochemical properties of the passive layer, potentiostatic tests were carried out for the analyzed samples (Figure 7). Before the test, the surface of the samples was properly prepared by polishing with sandpaper. The samples were immersed in a 3M aqueous NaCl solution and potentiostatically polarized in +700 mV SCE for 120 s at 25 °C. The reference electrode was a calomel electrode.

The steel specimen that had not been annealed showed excellent corrosion resistance—no current flow was recorded. As a result of heat treatment, the resistance to corrosive agents decreased. The higher the temperature of the annealing process, the higher the current values that were obtained, which means that the sample showed lower corrosion resistance. During annealing at a temperature of 600 °C, a decreasing trend in the function of current intensity from time is noticeable. This means a passive behavior of the sample resulting from a much smaller amount of σ phase than in the case of higher temperature treatment. Dependencies obtained for samples annealed at 800 °C are increasing functions (high final current was recorded), which indicates the weakest corrosion resistance among the compared steels.

## 4. Conclusions

This paper presented results investigating the effect of annealing on the structure and properties of duplex and super duplex steels. Several properties demonstrated by the samples in the state before and after heat treatment were compared. The primary conclusions are as follows:

1. The performed heat treatment, even under the least restrictive conditions (600 °C, 250 h), resulted in the precipitation inside the ferrite of the harmful intermetallic phase—the σ phase and the growth of austenite areas—and secondary austenite was formed. These structural changes resulted in an increase in the hardness of the samples.
2. During higher temperature annealing, the σ phase is less compared to the annealing effect at 600 °C. This is in line with the analysis of the Fe–Cr equilibrium diagram.
3. Long-term annealing (for 500 h) reduced the amount of the σ phase and in-creased the occurrence of dark ferrite areas. Most likely, the σ phase had partially dissolved and the recovering ferrite was ferrite δ. According to the Fe–Fe_3_C equilibrium diagram, ferrite δ is formed at 1394 °C. However, the high amount of alloying elements could reduce this temperature.
4. During the annealing of steels, a temperature of 800 °C was observed, leading to the disappearance of the banded structure (uniform structure). It can be due to this that the grain size became coarse, and banded austenite gradually evolved into the structure, which is similar to “island” and “bamboo”.
5. The loss of magnetic properties of both samples after long-term annealing, both at 600 and 800 °C, may result from the formation of a large amount of paramagnetic secondary austenite and probably also δ ferrite devoid of magnetic properties.
6. The decrease in corrosion resistance compared to the initial state was visible for all samples, and the material annealed at 600 °C was passivated. The samples annealed at a higher temperature (800 °C) showed worse corrosion resistance than the samples annealed at 600 °C. This is due to the transformation of ferrite into secondary austenite and precipitates of excess chromium in the form of the σ phase.
7. The conducted experiments have shown that the working temperature of duplex and super duplex steels must be clearly lower than 600 °C. The literature data define the operating temperature range for these steels from −50 to +300 or +500 °C. The operating temperature of most of the analyzed groups of apparatuses is below +500 °C. This confirms the possibility of using duplex steel as a construction material.

## Data Availability

The data presented in this study are available on request from the corresponding author.
